# Value of three‐dimensional speckle‐tracking imaging in detecting left ventricular systolic function in patients with dilated cardiomyopathy

**DOI:** 10.1111/echo.14427

**Published:** 2019-07-03

**Authors:** Jionghong He, Long Yang

**Affiliations:** ^1^ Department of Cardiology Guizhou Provincial People's Hospital Guiyang China

**Keywords:** dilated cardiomyopathy, echocardiography, left ventricular systolic function, magnetic resonance imaging, three‐dimensional speckle‐tracking imaging

## Abstract

**Objective:**

To explore the value of three‐dimensional speckle‐tracking imaging (3DSTI) in detecting left ventricular systolic function in patients with dilated cardiomyopathy (DCM).

**Methods:**

Totally 31 DCM patients were enrolled in this study. Left ventricular end‐systolic volume (LVESV), left ventricular end‐diastolic volume (LVEDV), and left ventricular ejection fraction (LVEF) were measured using the 3DSTI, two‐dimensional echocardiography (2DE), and magnetic resonance imaging (MRI). Left ventricular end‐diastolic mass (EDmass) and left ventricular end‐diastolic mass index (LVEDmass I) were also detected by 3DSTI and MRI. The differences in these measurements were analyzed and compared.

**Results:**

The values of LVESV, LVEDV, and LVEF showed significantly positive correlations among 2DE group, 3DSTI group, and MRI group. The LVEF value showed significant difference among these three groups [(33.3 ± 11.1)%, (30.3 ± 10.6)%, and (26.2 ± 10.7)%; *P *=* *0.04], whereas LVEDV and LVESV values were not significantly different (*P *&gt; 0.05; respectively). Inter‐group comparison showed the mean of LVEF was significantly lower in MRI group than in 2DE group (*P *=* *0.031), whereas there was no significant difference between 2DE group and 3DSTI group and between 3DSTI group and MRI group (*P *&gt; 0.05; respectively). The EDmass and EDmassI detected by 3DSTI and MRI were (143.2 ± 40.2) g vs (190.0 ± 58.3) g and (83.2 ± 21.1) g/m^2^ vs (110.1 ± 29.7) g/m^2^ (*P *<* *0.001; respectively).

**Conclusions:**

The LVEF value detected by 3DSTI is closer to that detected by MRI in DCM patients.

## 1 INTRODUCTION

Three‐dimensional speckle‐tracking imaging (3DSTI) can track the motion of the speckles in a real time and angle‐independent manner and thus can evaluate left ventricular (LV) function more comprehensively and accurately.^1^, ^2^, ^3^, ^4^, ^5^, ^6^, ^7^ However, the role of 3DSTI, two‐dimensional echocardiography (2DE), and magnetic resonance imaging (MRI) in detecting left ventricular ejection fraction (LVEF) in patients with primary dilated cardiomyopathy (DCM) remains controversial.

## 2 SUBJECTS AND METHODS

### 2.1 Subjects

A total of 31 DCM patients (27 males [87.1%] and 4 females [12.9%] aged 32–74 years ([52.8 ± 10.9] years) were enrolled in this study. These patients were in New York Heart Association (NYHA) class III–IV.

### 2.2 Equipment

The equipment used in this study included: a) the Vivid E9 Ultrasound System (GE Healthcare), which used the M3S and V3 probes with frequencies of 1.7–3.4 and 1.7–3.5 MHz, respectively, for 3DSTI and used the M5S probe with frequencies of 2.0–4.5 MHz for 2DE. Cardiac MRI was performed using a 1.5T MR scanner (Siemens Healthcare) with a 48‐channel phased array surface coil.

### 2.3 Methods

Cardiac images were collected and stored by 2DE and 3DSTI, respectively. The images were acquired by the same cardiac sonographer and then the 2DE and 3DSTI images were analyzed independently by two other cardiac sonographers respectively. Each image was analyzed twice by the same cardiac sonographer at different times, and the average value of the two values is used for statistical analysis.

#### 2.3.1 2DE

Left ventricular end‐systolic volume (LVESV), left ventricular end‐diastolic volume (LVEDV), and LVEF were measured using the biplane Simpson's method in the apical two‐ and four‐chamber views.

#### 2.3.2 3DSTI

The M3S probe was placed at the apex and near the sternum to collect the 2D ultrasound images in four‐chamber, two‐chamber, left ventricular long‐axis, mitral valve level, papillary muscle level, and apical left ventricular short‐axis views. The V3 probe was placed at the apex to collect images in the apical four‐chamber, two‐chamber, and left ventricular long‐axis views, followed by collection of four consecutive cycles of cone images when patients were holding their breath to form the left ventricular full volumetric 3D images. The images were processed and stored by using the GE EchoPac software. The full volumetric images during the systolic and diastolic periods in the four‐chamber, two‐chamber, and left ventricular long‐axis views were collected to determine the endpoints of the connection line between apex and mitral annulus. The software automatically generated the left ventricular endocardial and the epicardial boundaries, which could be manually adjusted. Subsequently, the software automatically generated LVESV, LVEDV, LVEF, and left ventricular end‐diastolic mass (EDmass). Then, the left ventricular end‐diastolic mass index (LV EDmass I) was calculated based on the body height and body weight.

#### 2.3.3 MRI

The images at transverse, coronal, and sagittal positions and in the standard two‐ and four‐chamber views were collected; with these images as scout images, the cine images in short‐axis, two‐chamber, and four‐chamber views were acquired. A fast gradient echo pulse sequence was used to scan from the base of the heart to the apex sequentially, and the results were collected 6–8 times. The scanning parameters were as follows: time of repetition (TR), 39.75 ms; time of echo (TE), 1.11 ms; layer thickness, 8 mm; interlayer spacing, 0 mm; field of vision (FOV), 340 × 276 mm; deflection angle, 80°; and bandwidth, 930 Hz. After the scanning was completed, the original images were input into the postprocessing workstation, and the epicardium and endocardium regions were delineated from the base of the heart to the apex by manual sketching, and the results were adjusted by manual and semiautomatic calibration. The software automatically calculated LVESV, LVEDV, LVEF, and EDmass. Then, the LV EDmass I was calculated based on the body height and body weight.

### 2.4 Statistical analysis

Data were expressed as mean ± standard deviations (*x *± *s*). *One‐way* ANOVA was used to analyze the data differences among these three groups, and *Student‐Newman‐Keuls test* was used to compare the data between two groups. The comparisons between two groups were based on *t* test. Correlation analysis was based using *Pearson correlation analysis*;* P *<* *0.05 was considered significantly different.

## 3 RESULTS

### 3.1 Comparisons of left ventricular function parameters

**Figure 1:**
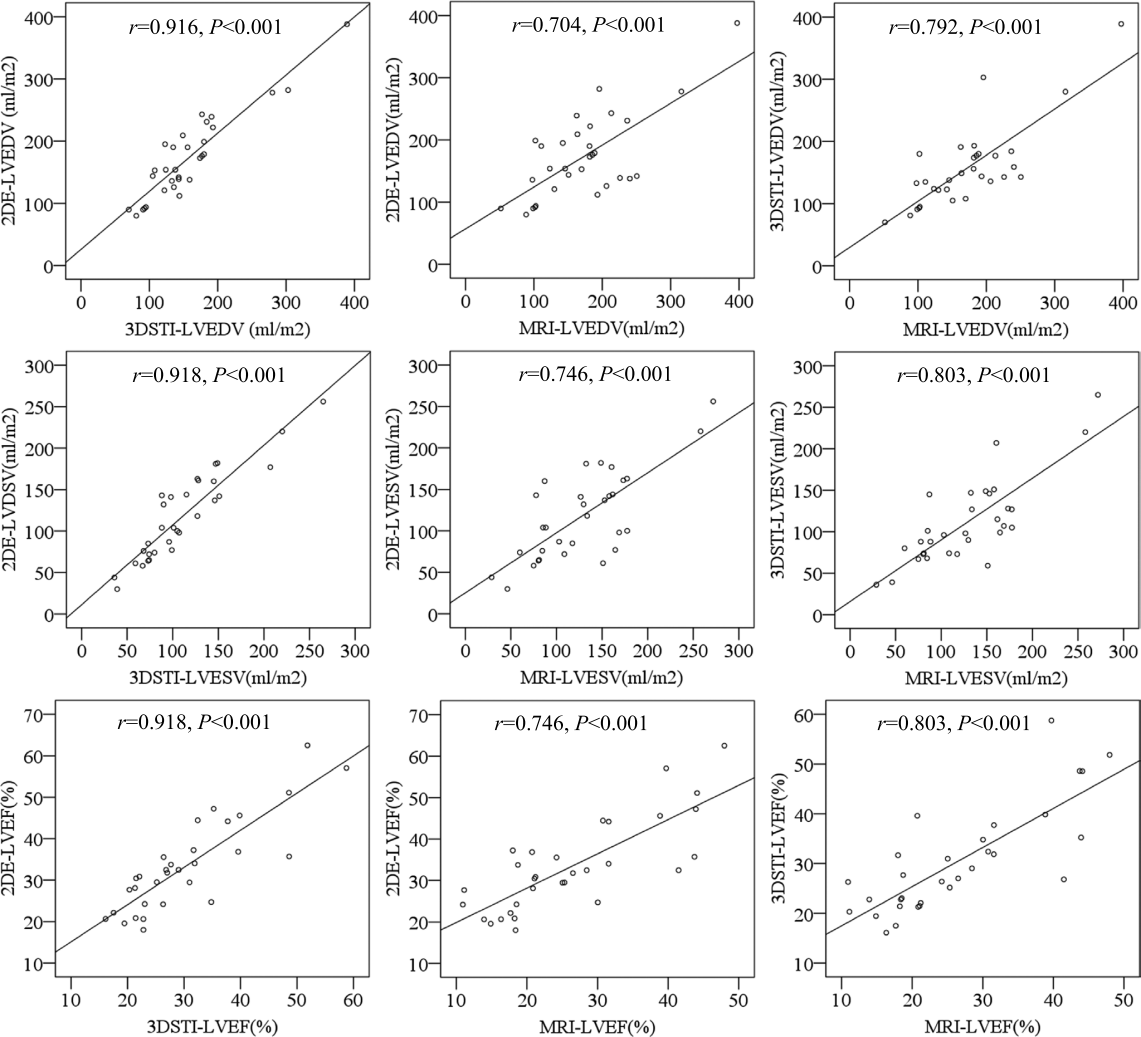
The results of *Pearson* correlation analysis among the left ventricular systolic function parameters (n* *=* *31)

The values of LVESV, LVEDV, and LVEF detected by the three methods were positively correlated with each other (*P *<* *0.001, respectively; Figure 1). The mean of LVEF was largest in the 2DE group, followed by the 3DSTI group and the MRI group, with a significant difference (*P *<* *0.05; Table 1); inter‐group comparison showed the mean of LVEF was significantly different between 2DE group and MRI group (*P *<* *0.05), whereas there was no significant difference between 2DE group and 3DSTI group and between 3DSTI group and MRI group (*P *>* *0.05, respectively). The means of LVESV and LVEDV showed no significant difference among three groups (*P *>* *0.05, respectively).

**Table 1:** Comparison of the left ventricular systolic function parameters (*x* ± *s*, n* *=* *31)

	2DE	3DSTI	MRI	*P*
LVESV(mL/m^2^)	117.9 ± 53.6	111.0 ± 51.1	128.0 ± 55.2	0.455
LVEDV(mL/m^2^)	172.9 ± 67.8	157.3 ± 66.4	172.1 ± 70.9	0.601
LVEF (%)	33.3 ± 11.1	30.3 ± 10.6	26.2 ± 10.7*	0.040

*P =* *0.031 compared with that in 2DE group.

### 3.2 Comparisons of left ventricular mass

The EDmass and EDmassI detected by 3DSTI and MRI were (143.2 ± 40.2) g vs (190.0 ± 58.3) g and (83.2 ± 21.1) g/m^2^ vs (110.1 ± 29.7) g/m^2^, decreased by 24.6% and 24.4% (*P *< 0.001, respectively). Therefore, the left ventricular diastolic myocardial mass measured by 3DSTI was underestimated compared with that measured by MRI.

## 4 DISCUSSION

Left ventricular ejection fraction is the most valuable indicator that reflects the degree of left ventricular systolic dysfunction and predicts the prognosis of patients with chronic congestive heart failure (CHF). LVEF is calculated as the difference between LVEDV and baseline LVEDV, divided by baseline LVEDV. Accurate measurement of LVESV and LVEDV ensures a true LVEF.

The modified Simpson method is the most commonly used method for detecting LVEF in clinical settings. It estimates LVESV and LVEDV by measuring intracardiac area. Its accuracy is based on the uniformity and coordination of the left ventricular wall motion, and there is no abnormal segmental wall motion. In theory, the LVESD and LVEDV yielded by 3DSTI are based on the change in left ventricular volume and thus are more accurate than the LVESV and LVEDV values estimated by 2DE, especially when the left ventricular wall motion is uncoordinated or segmental.^8^, ^9^ It has been reported that 3DSTI has obvious advantages in evaluating left ventricular volume and cardiac function in patients with coronary heart disease and myocardial infarction.^10^ Pathologically, DCM is based on abnormal myocytes and often manifests as diffuse weakening of left ventricular motion. Theoretically, compared with patients with uncoordinated wall motion, the LVEF value obtained by 2DE in DCM patients is closer to that measured by 3DSTI.

As shown in our current study, there were significant positive correlations among the values of LVESV and LVEDV obtained by 2DE, 3DSTI, and MRI and then the LVEF value, suggesting the data obtained by these three methods are quite similar. Currently, the MRI is regarded as the gold standard for detecting left ventricular systolic function. Therefore, the values of LVESV, LVEDV, and LVEF obtained by both 2DE and 3DSTI can, at a certain degree, reflect the real left ventricular systolic function. Although there was no significant difference in the values of LVESV and LVEDV among these three groups (*P *>* *0.05), the values of LVEF, calculated by LVESV and LVEDV, showed significant difference among these three groups (*P *<* *0.05). The LVEF values showed a decreasing trend in 2DE, 3DSTI, and MRI groups. Although the LEVF value in 3DSTI group was not significantly different from those in 2DE group and MRI group (*P *>* *0.05, respectively), it was significantly different between MRI group and 2DE group (*P *<* *0.05). In DCM patients, the value of LVEF obtained by 3DSTI was closer to that obtained by MRI than that obtained by 2DE. Of course, only 31 cases of DCM patients were included in this study, which may be the reason why there was no statistical difference in LVEF measurement between 3DSTI group and 2DE group.

For CHF patients, the left ventricular myocardial mass reflects the degree of left ventricular remodeling and is an indirect indicator of left ventricular dysfunction. In our current study, the mean values of EDmass and EDmassI in 3DSTI group were 24.6% and 24.4% lower than those measured by MRI (*P *<* *0.001, respectively), suggesting that the results of left ventricular end‐diastolic myocardial mass measured by 3DSTI were remarkably underestimated when compared with those in MRI.

In conclusion, LVEF is the most commonly used parameter for evaluating left ventricular systolic function. In DCM patients, LVEF value measured by 3DSTI is closer to that detected by MRI. It indicated that, as a more convenient and affordable technique on LVEF detection, 3DSTI is more feasible in clinical application than MRI.

## References

[1] Xu TY , Sun JP , Lee AP , et al. Three‐dimensional speckle strain echocardiography is more accurate and efficient than 2D strain in the evaluation of left ventricular function. Int J Cardiol. 2014;176:360–366. 25088155 10.1016/j.ijcard.2014.07.015

[2] Luo Y , Liu Y , Guan X , et al. Value of three dimensional‐speckle tracking imaging for predicting left ventricular function after non‐ST‐segment elevation myocardial infarction with percutaneous coronary intervention. J Xray Sci Technol. 2018;26:331–339. 29562571 10.3233/XST-17316

[3] Lorenzini C , Lamberti C , Aquilina M , et al. Reliability of left ventricular ejection fraction from three‐dimensional echocardiography for cardiotoxicity onset detection in patients with breast cancer. J Am Soc Echocardiogr. 2017;30:1103–1110. 28822666 10.1016/j.echo.2017.06.025

[4] Tao ZW , Ma XW , Liu NN , et al. Evaluation on the impact of spontaneous reperfusion on cardiac muscle of acute myocardial infarction by three‐dimensional speckle tracking imaging. Eur Rev Med Pharmacol Sci. 2017;21:5445–5450. 29243788 10.26355/eurrev_201712_13933

[5] Balasubramanian S , Punn R , Smith SN , et al. Left ventricular systolic myocardial deformation: a comparison of two‐ and three‐dimensional echocardiography in children. J Am Soc Echocardiogr. 2017;30:974–983. 28802483 10.1016/j.echo.2017.06.006

[6] Bhaya M , Sudhakar S , Sadat K , et al. Effects of antegrade versus integrated blood cardioplegia on left ventricular function evaluated by echocardiographic real‐time 3‐dimensional speckle tracking. J Thorac Cardiovasc Surg. 2015;149:877–884. 25623902 10.1016/j.jtcvs.2014.11.034

[7] Luis SA , Yamada A , Khandheria BK , et al. Use of three‐dimensional speckle‐tracking echocardiography for quantitative assessment of global left ventricular function: a comparative study to three‐dimensional echocardiography. J Am Soc Echocardiogr. 2014;27:285–291. 24325960 10.1016/j.echo.2013.11.002

[8] van Dalen BM . Left ventricular ejection fraction by real‐time three‐dimensional echocardiography: the Necker cube for the naive realism of two‐dimensional methods. Neth Heart J. 2014;22:380–382. 25112790 10.1007/s12471-014-0579-z PMC4160458

[9] Xu Y , Shi J , Zhao R , et al. Anthracycline induced inconsistent left ventricular segmental systolic function variation in patients with lymphoma detected by three‐dimensional speckle tracking imaging. Int J Cardiovasc Imaging. 2019;35:771–779. 30684081 10.1007/s10554-018-1510-2

[10] Marsan NA , Westenberg JJ , Roes SD , et al. Three‐dimensional echocardiography for the preoperative assessment of patients with left ventricular aneurysm. Ann Thorac Surg. 2011;91:113–121. 21172497 10.1016/j.athoracsur.2010.08.048

